# Microglial AGE-Albumin Is Critical in Promoting Alcohol-Induced Neurodegeneration in Rats and Humans

**DOI:** 10.1371/journal.pone.0104699

**Published:** 2014-08-20

**Authors:** Kyunghee Byun, Delger Bayarsaikhan, Enkhjargal Bayarsaikhan, Myeongjoo Son, Seyeon Oh, Jaesuk Lee, Hye-in Son, Moo-Ho Won, Seung U. Kim, Byoung-Joon Song, Bonghee Lee

**Affiliations:** 1 Center for Genomics and Proteomics, Lee Gil Ya Cancer and Diabetes Institute, Gachon University, Incheon, Korea; 2 Department of Anatomy and Cell Biology, Gachon University Graduate school of Medicine, Incheon, Korea; 3 Department of Bioengineering, University of California, Berkeley, CA, United States of America; 4 Department of Anatomy and Neurobiology, and Institute of Neurodegeneration and Neuroregeneration, College of Medicine, Kangwon National University, Chuncheon, Korea; 5 Department of Medicine, University of British Columbia, Vancouver, Canada; 6 Laboratory of Membrane Biochemistry and Biophysics, National Institute on Alcohol Abuse and Alcoholism, National Institutes of Health, Bethesda, MD, United States of America; Instituto Murciano de Investigación Biosanitaria-Virgen de la Arrixaca, Spain

## Abstract

Alcohol is a neurotoxic agent, since long-term heavy ingestion of alcohol can cause various neural diseases including fetal alcohol syndrome, cerebellar degeneracy and alcoholic dementia. However, the molecular mechanisms of alcohol-induced neurotoxicity are still poorly understood despite numerous studies. Thus, we hypothesized that activated microglial cells with elevated AGE-albumin levels play an important role in promoting alcohol-induced neurodegeneration. Our results revealed that microglial activation and neuronal damage were found in the hippocampus and entorhinal cortex following alcohol treatment in a rat model. Increased AGE-albumin synthesis and secretion were also observed in activated microglial cells after alcohol exposure. The expressed levels of receptor for AGE (RAGE)-positive neurons and RAGE-dependent neuronal death were markedly elevated by AGE-albumin through the mitogen activated protein kinase pathway. Treatment with soluble RAGE or AGE inhibitors significantly diminished neuronal damage in the animal model. Furthermore, the levels of activated microglial cells, AGE-albumin and neuronal loss were significantly elevated in human brains from alcoholic indivisuals compared to normal controls. Taken together, our data suggest that increased AGE-albumin from activated microglial cells induces neuronal death, and that efficient regulation of its synthesis and secretion is a therapeutic target for preventing alcohol-induced neurodegeneration.

## Introduction

Alcohol (ethanol), being widely-used in human societies, is one of the most well-known neurotoxic agents, since long-term heavy consumption of alcohol causes injury to many tissues including liver, pancreas, brain, etc [1]. In the brain, heavy alcohol ingestion promotes abnormal behavior and disorders of the central nervous system [2]–[4]. It has been experimentally demonstrated that sub-chronic exposure of alcohol (e.g., 5 g/kg/day oral gavage for 10 days) causes neuronal loss in certain brain areas such as the hippocampus and entorhinal cortex [5]–[10].

Alcohol consumption also induces neuroinflammation, which then activates microglia. In fact, these regions of alcoholic brain injury also display elevated appearance of activated microglial cells [11]–[14]. The relationship between alcohol consumption and microglial activation has long been studied, but their pathological roles have been poorly established [15]–[18].

It is well-established that alcohol consumption results in significant accumulations of monocyte chemoattractant protein-1 (MCP-1, CCL2) in the ventral tegmental area, substantia nigra, hippocampus, limbic associated regions, and amygdala of alcoholic brains [15], [19]–[22]. CYP2E1 has also been reported in the same areas after ethanol treatment, and these are key molecules that activate microglia [23]–[25]. However, the molecular mechanism for ethanol-induced neurotoxicity by activating microglia is poorly understood. Thus, we hypothesized that AGE-albumin from microglial cells activated by elevated MCP-1 or CYP2E1 and its receptor of advanced glycation end product (RAGE) could promote neuronal death in the same regions where MCP-1 is accumulated following ethanol exposure.

The aim of this study was to study the relationship among ethanol consumption, microglial activation, and neuronal death in specific areas of the brain through increased AGE-albumin and RAGE. In addition, we aimed to study whether inhibition of AGE and RAGE protects against alcohol-induced neuronal death in both in vitro and animal models.

## Materials and Methods

### Cell culture

Immortalized human microglial cells (HMO6) and human neuroblastoma cells (SH-SY5Y) were used for the *in vitro* studies [26]–[28]. HMO6 and SH-SY5Ycells were grown in Dulbecco's modified Eagle's medium (Gibco) containing high glucose concentration and supplemented with 10% fetal bovine serum (Gibco) and 20 mg/ml gentamycin (Sigma-Aldrich) at 37°C under 5% CO_2_. HMO6 cells were exposed to ethanol (Dukan) at concentrations of 25, 50, and 100 mM for 24 hours. SH-SY5Y cells were harvested after 24 hour AGE-ALB (50 mM, Sigma-Aldrich) treatment for cell death mechanism analysis.

### Human brain tissues

Brain tissues from normal control and alcoholic individuals were obtained from the National Pathology Center of Mongolia. Human brain samples were collected from cadavers with history of chronic alcoholism. Tissues were collected from cortex and directly fixed in 10% formalin buffer overnight and transferred to automatic dehydration system (Leica ASP-300 S). Dehydration steps consists of 90% ethanol −3 times each for 1 hour at RT and 100% ethanol −2 times each for 2 hours at RT. After dehydration, Tissues were cleared with xylene −1 time for 1.5 hour and embedded in paraffin at 60°C. The information of the patients (n = 5/group) is listed in Table 1. The brain tissue collection and usage were approved by the Ethics Committee of the Mongolian National Cancer Center (#BZ26-2013), Ulaanbaatar, Mongolia.

**Table 1:** List of characteristics of human specimens including alcoholic individuals (1–5) and control (6–10).

No.	Age	Gender	Disease classification
1	26	Male	Alcoholic hepatitis
2	38	Male	Liver cirrhosis
3	42	Male	Liver cirrhosis
4	43	Male	Liver cirrhosis
5	72	Male	Liver cirrhosis
6	50	Male	Normal
7	53	Male	Normal
8	44	Female	Normal
9	77	Male	Normal
10	53	Female	Normal

### Immunoblot analysis

Total cell lysates were prepared with lysis buffer (7 M urea (Amresco), 2 M thiourea (Amresco), 4% CHAPS (3-[(3-Cholamidopropyl) dimethylammonio]-1-propanesulfonate, Amresco), and 5 mM DTT (dithiothreitol, Amresco), pH = 7.6) followed by sonication. The lysates were centrifuged at high speed of 17,000 g for 20 mins at 4°C. Total protein concentration was measured by the QUBIT method. Equal amounts of proteins were separated on polyacrylamide gels and transferred to nitrocellulose membranes for immunoblot analysis. The transferred proteins on nitrocellulose membrane were detected with the protein-specific antibodies followed by the secondary antibodies, as described [28].

### Co-immunoprecipitation

Total cell lysates were prepared in lysis buffer (1 M Tris (pH 7.5, Amresco), 5 M NaCl (Sigma aldrich), 10% NP-40 (Fluka), 10% deoxycholate and protease inhibitor cocktail (Roche)) followed by sonication. The lysates were centrifuged at 17,000×g for 20 min, and the supernatant was incubated overnight with the respective primary antibody at 4°C under constant head-to-tail rotation. Immunoprecipitates were collected with Protein G agarose beads. The beads were washed in a lysis buffer (1 M Tris (pH 7.5, amresco), 5 M NaCl (Sigma aldrich), 10% NP-40 (Fluka), 10% deoxycholate and protease inhibitor cocktail (Roche)), and the immunoprecipitates were resuspended in 1×SDS sample buffer. Total protein concentration was measured by the QUBIT method according to the manufacturer's method. Equal amounts of protein were separated on polyacrylamide gels and transferred to nitrocellulose membranes for immunoblot analysis for the target proteins, as indicated.

### Enzyme-linked immunosorbent assay (ELISA)

Wells of a 96-well microplate were coated with 1 µg/ml of albumin antibody in 100 mM carbonate/bicarbonate buffer (pH 9.6) overnight at 4°C. After washing the wells twice with PBS, the remaining protein-binding sites were blocked by adding 5% skim milk (Sigma-Aldrich) at 4°C overnight. After washing with PBS, the samples of different tissue extracts were added into the wells and incubated for 90 min at 37°C. After rinsing with PBS, 1 µg/ml of AGE antibody (Abcam) was added for 2 h at room temperature. After washing the plate with PBS, the samples were incubated for 2 h at room temperature with horse radish peroxidase-conjugated secondary antibody (Vector Laboratories). After adding the substrate TMB solution (Sigma-Aldrich) followed by incubated for 30 min, an equal volume of stop solution (2 N H_2_SO_4_) was added, and optical density was determined at 450 nm. Each sample was repeated 5 times (Table S.1).

### Alcohol exposed rodents

Sprague–Dawley rats (weighing 250–300 g) were sub-chronically treated with alcohol by following the previous methods [10], [29]. Briefly, ethanol (25% v/v) was administered orally by 5 g/kg once a day for 10 days. Co-treatment group (alcohol and pyridoxamine) received 1 g/L concentration pyridoxamine in drinking water from one day before alcohol treatment for 11 days. All animal experiments were approved by the Institute Animal Care and Use Committee of Lee Gil Ya Cancer and Diabetes Institute of Gachon University (#LCDI-2012-0073) and conducted humanely.

### Brain tissue preparation

Animals were sacrificed under anesthesia and treated transcardially with 50 ml PBS followed by 150 ml cold fixative consisting of 4% paraformaldehyde (PFA) in PBS. After perfusion, the brains were removed and post-fixed for 5 h in 4% PFA followed by soaking in 20% sucrose solution overnight. The cryoprotected brains were cut into 10 µm slices using a cryotome (Leica, Wechsler, Germany).

### Immunostaining

Rat brain slices of different treatments were rinsed with cold distilled water and Triton X100-PBS. Normal serum was used to block non-specific antibody binding. Frozen sections of rat brains were washed five times in 1X PBS at the same time and incubated with the primary antibody specific to each target protein. After overnight incubation with the primary antibodies (Table 2) at 4°C, the excess antibodies were washed with PBS, followed by incubation with a secondary antibody at room temperature for 1 h to obtain images. Nuclei were counterstained in DAPI (4′6-diamino-2-phenilindole; 1 µg/ml, Sigma-Aldrich) for 20 seconds. After washing with PBS, coverslides were mounted using Vectashield mounting media (Vector Laboratories) and images analyzed using a LSM 710 confocal microscope at the same setting and same time (Carl Zeiss, Oberkochen, Germany).

**Table 2:** List of antibodies used in this study.

Antigen	Host	Company (Cat No)	Application IHC WB	
Albumin	Mouse	Abcam (ab10241)	1∶100	1∶1000
AGE	Rabbit	Abcam (ab23722)	1∶200	1∶3000
Iba-1	Goat	Abcam (ab5076)	1∶100	1∶1000
RAGE	Goat	Abcam (ab7764)	1∶400	1∶4000
OX-42	Mouse	Millipore (CBL1512F)	1∶50	
p38	Rabbit	Cell signaling (9212L)	1∶100	1∶1000
pp38	Rabbit	Cell signaling (9211S)	1∶100	1∶1000
ERK1/2	Rabbit	Cell signaling (9102S)	1∶100	1∶1000
pERK1/2	Rabbit	Cell signaling (4377S)	1∶100	1∶1000
SAPK/JNK	Rabbit	Cell signaling (9252S)	1∶100	1∶1000
pSAPK/JNK	Rabbit	Cell signaling (9251S)	1∶100	1∶1000
β-Actin	Rabbit	Abcam (ab8227)	1∶100	1∶1000
Peroxidase labeled anti-mouse IgG	Mouse	Vector (PI2000)		1∶5000
Peroxidase labeled anti-rabbit IgG	Rabbit	Vector (PI 1000)		1∶5000
Peroxidase labeled anti-goat IgG	Goat	Vector (PI9500)		1∶5000
Alexa Fluor 555 donkey anti rabbit IgG	Rabbit	Invitrogen (A31572)	1∶500	
Alexa Fluor 633 goat anti rabbit IgG	Rabbit	Invitrogen (A21070)	1∶500	
Alexa Fluor 555 donkey anti goat IgG	Goat	Invitrogen (A21432)	1∶500	
Alexa Fluor 488 donkey anti mouse IgG	Mouse	Invitrogen (A11001)	1∶500	

### DAB (3,3′-diaminobenzidine) staining and cell counting

All tissue slides were washed in 1XPBS five times and immersed into 0.3% H_2_O_2_ for 15 mins at room temperature to reduce endogenous peroxidase activity. Tissues were washed in 1XPBS three times and incubated with primary antibody (OX-42) at 4°C overnight. The tissues were washed again with 1XPBS. ABC solution (Vector laboratories) was added to tissues and incubated at room temperature for 1 hr. After washing with 1XPBS, biotinylated secondary antibody was incubated for 1 hr at room temperature. Tissues were washed again in 1XPBS three times and incubated with DAB (Sigma-Aldrich) solution at room temperature for 1 min. Finally tissues were washed in a graded ethanol series (70% 30 sec, 80% 30 sec, 95% 30 sec, 100% 30 sec and 100% Xylene 5 min). The stained slides were mounted with DPX mounting medium (Sigma-Aldrich) for microscopic image analysis. The numbers of the activated microglial cells in areas of CA1, CA2, CA3 or dendate gyrus were counted under 200× magnification by three different researchers separately, and the total number was generated by adding each number. Then average numbers of each areas were analyzed statistically.

### Cresyl violet staining

Frozen rat brain sections (10 µm) were washed five times in 1X PBS and hydrated in a graded ethanol series (100% ethanol 5 min, 95% ethanol 5 min, 70% ethanol 5 min, 50% and water 30 sec). After washing with distilled water, the tissue slices were stained with 0.5% cresyl violet acetate (Sigma-Aldrich) solution for 8 mins and washed in a graded ethanol series (70% 30 sec, 80% 30 sec, 95% 30 sec, 100% 30 sec and 100% Xylene 5 min). The stained slides were mounted with DPX mounting medium (Sigma-Aldrich) for microscopic image analysis. Three researchers counted cell number with masked (blind) way. Each researcher counted 5 sections containing hippocampus per animal and analyzed statistically (Table S. 2).

### TUNEL assay

Frozen rat brain tissues were washed 3 times in 1XPBS and incubated with permeabilization solution (0.1% Trinton X-100, 0.1% Sodium citrate, freshly prepared) 2 mins on ice. Then tissues were rinsed 3 times with 1XPBS. TUNEL reaction mixture (Roche) was added on tissues and incubated in a humidified atmosphere for 1 hr at 37°C in the dark. Tissues were washed again in 1XPBS 3 times and coverslips were mounted onto glass slides using the Vectashield mounting medium (Vector Laboratories). Apoptotic cells were analyzed under fluorescent microscope (Zeiss Imager.Z1).

### Densitometry and statistical analysis

The densitometric intensity of each immunoreactive band was determined using Image-Pro gel digitizing software. All data shown in this study represent results from at least three independent experiments. Statistical analyses were performed using the Student's *t*-test and a *p*<0.05 was considered significant (*; p<0.05, **; p<0.001, ***; p<0.0001).

## Results

### 1. Ubiquitous distribution of AGE-albumin and activated microglial cells in the brain of human alcoholics and the binge alcohol-exposed rats

**Figure 1:**
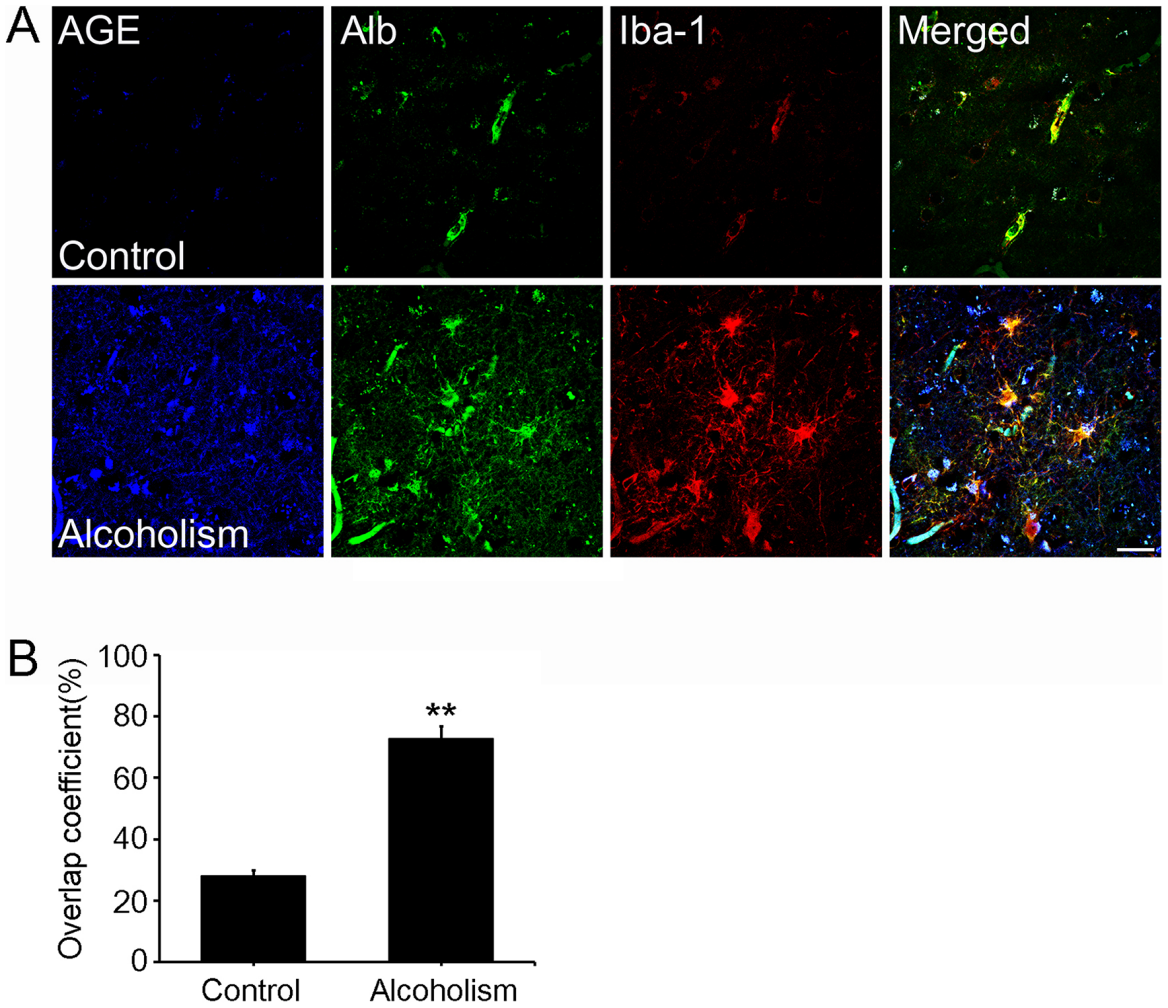
Distribution of AGE-albumin and activated microglial cells in the brains of human alcoholics and control people. (A) Triple-labeled confocal microscopic image analyses were used to study the distribution and relative levels of AGE (blue), albumin (green), and Iba-1 (red) in the cerebral cortex of human brains from normal people or alcoholic individuals. (B) Densitometry analyses of AGE, albumin, and Iba-1 colocalization were evaluated using Zeiss Zen software. Scale bar  = 50 µm. **p<0.001.

We first investigated the respective distribution of AGE and albumin in the brains of human alcoholics and normal individuals to study the mechanisms by which AGE-albumin was increased and how it promoted neuronal cell death. Surprisingly, most AGEs were co-localized with albumin and detected in activated microglial cells, suggesting that AGE-albumin could be a major AGE product in activated microglial cells in the brain of human alcoholics (Fig. 1A). Densitometric analysis indicated a dramatic increase in AGE-albumin in the brains of alcoholics (n = 5) compared to those from normal individuals (n = 5) (Fig. 1B).

**Figure 2:**
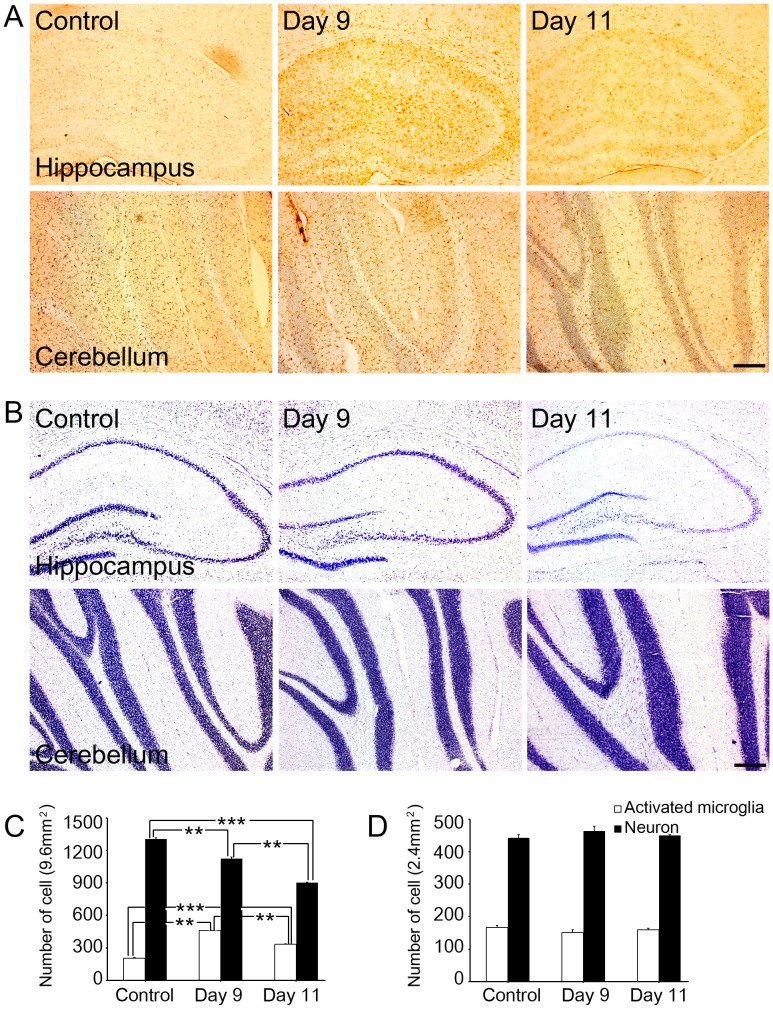
Distribution of activated microglial cells and neuron in the hippocampus of the control or rats exposed to binge-alcohol at days 9 and 11. (A) OX-42-positive microglial cells were measured by immunohistochemical staining in control or alcohol-exposed rats brains. (B) Cresyl violet staining was used to detect decreasing neuronal cell number in the hippocampus of alcohol-exposed rats. (C) Number of OX-42 positive and cresyl violet stained cells in the hippocampus in control or alcohol-exposed rats. (D) Number of OX-42 positive and cresyl violet stained cells in the cerebellum at control or alcohol-exposed rats (white bar; OX-42 positive cell (activated microglia), black bar; cresyl violet positive cell (neuron)). Scale bar  = 200 µm. **; p<0.001, ***; p<0.0001.

The immunohistochemical analysis of rat brain slices before and after alcohol treatment from day 1 to 11 revealed that the number of activated microglial cells increased significantly up to day 9 in the hippocampus of the binge ethanol-exposed rats, however the number of activated microglial cells decreased significantly at day 11 (Fig. 2A, C). Activated microglial cells also increased in ENT and VTA, but not in PAG of binge-ethanol model (Fig. S1A). Cresyl violet staining showed that the number of neuronal cells decreased maximally in the hippocampus of the alcohol-exposed rat brain on day 11 (Fig. 2B). Neuronal death was also observed in the ENT and VTA but not in PAG of binge-ethanol rat brain (Fig. S1B). In contrast, binge alcohol exposure did not alter the number of neuronal cells in the cerebellum (Figs. 2B, D).

**Figure 3:**
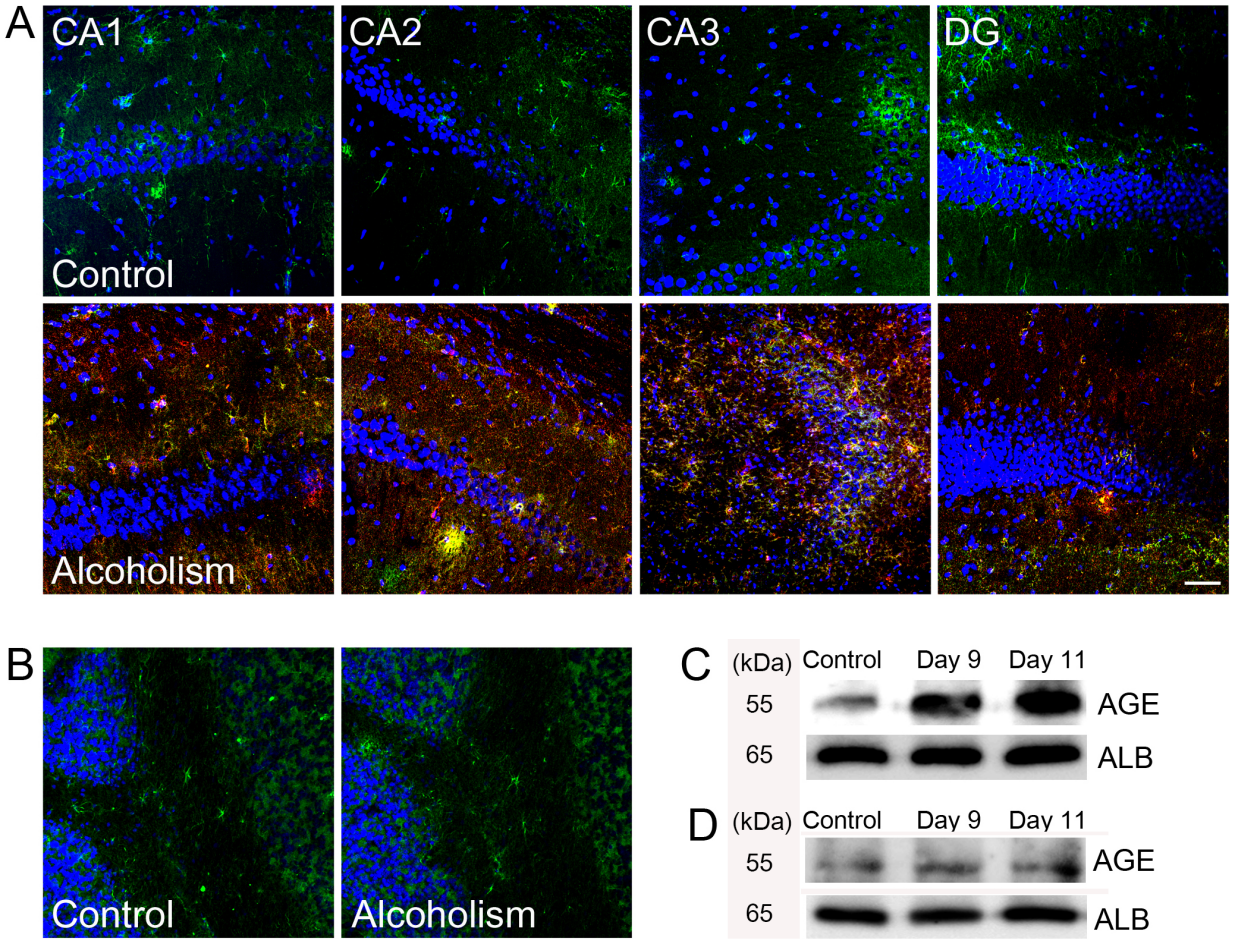
AGE-albumin level in rat brains. Triple-labeled confocal microscopic image analyses were used to study the distribution and relative levels of albumin (green), AGE (red), and DAPI (blue) in the hippocampus Cornu Ammonis Area1 (CA1), Cornu Ammonis Area2 (CA2), Cornu Ammonis Area3 (CA3), Dendate Gyrus (DG) of the control rats (A) and binge-alcohol exposed rats (B). Scale bar  = 50 µm. (C, D) Co-immuonoprecipitation analysis of AGE-ALB in the hippocampus (C) and cerebellum (D) in control or alcohol-exposed rats on days 9 and 11. Scale bar  = 50 µm.

In addition, tissue levels of AGE and albumin were markedly elevated, and AGE was co-localized with albumin in the hippocampus of the binge ethanol-exposed rat brains compared with that in control rats, as determined by triple-labeled confocal microscopic image analyses (Figs. 3A, Fig. S2). However, similar to the neuronal population, no obvious increase in AGE or albumin was detected in the periaqueductal grey area or cerebellum in the binge ethanol-exposed rat brains (Fig. 3B). Western blot analysis revealed that AGE-albumin level was increased in the hippocampus without significant change in the cerebellum at day 11 of the binge alcohol-exposed rats compared to control rats (Figs. 3C, D).

### 2. Synthesis and secretion of AGE-albumin in microglial cells following ethanol treatment

**Figure 4:**
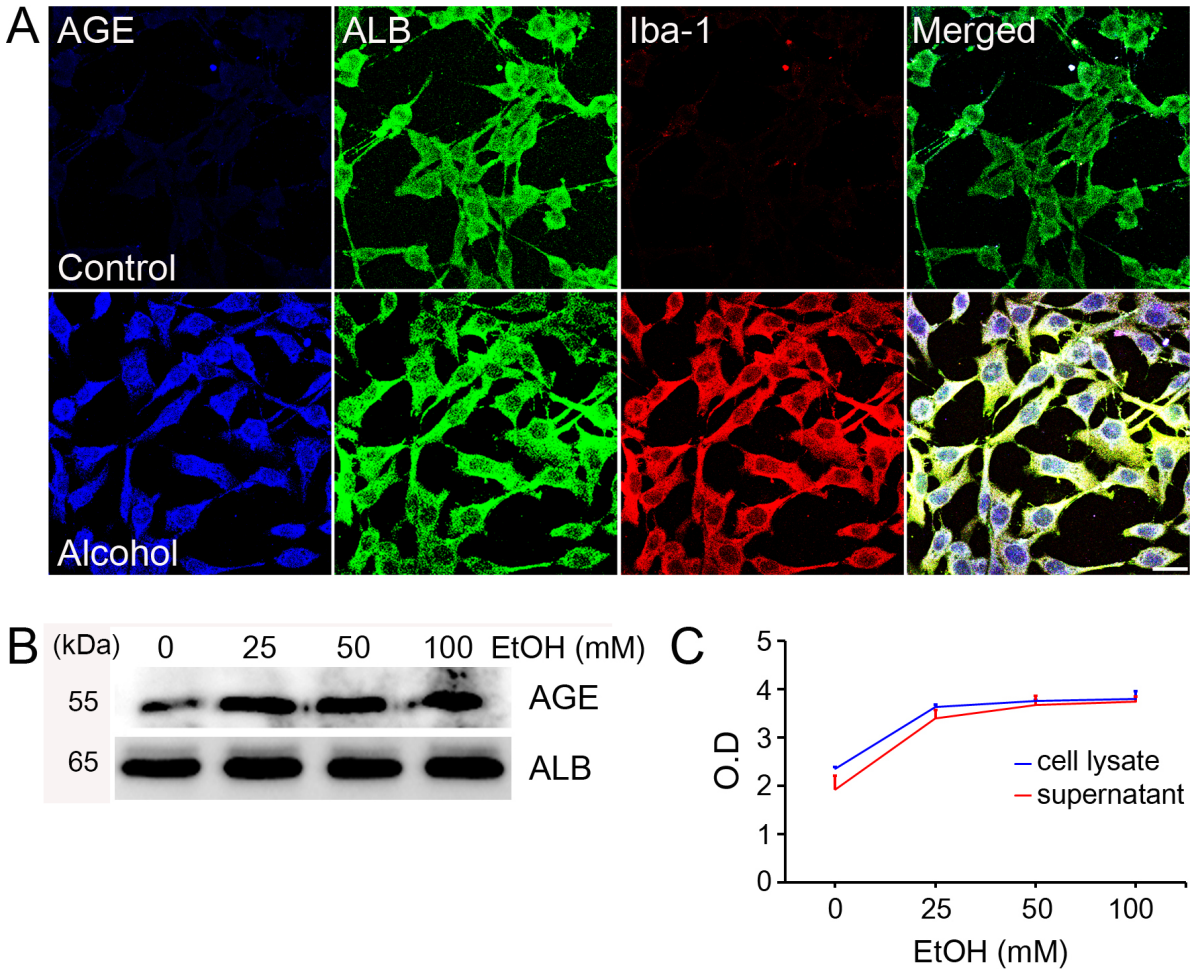
Increased synthesis of AGE-albumin in human microglial cells and its extracellular secretion. (A) Triple-labeled confocal microscopic image analyses were used to study the distribution and relative levels of AGE (blue), albumin (green), and Iba-1 (red) in human microglial cells after alcohol treatment. (B) The dose-dependent increments of AGE-albumin in total lysates of HMO6 cells treated with 0, 25, 50 or 100 mM ethanol for 24 h were determined by co-immunoprecipitation. (C) Graph illustrating the dose-dependent changes in intracellular (cell lysates) and extracellular (culture supernatant) levels of AGE-albumin in HMO6 cells treated with 0, 25, 50 or 100 mM ethanol for 24 hr, as determined by ELISA. Scale bar  = 50 µm.

Because of the elevated amounts of AGE-albumin in the hippocampus of rats compared to their respective controls, we further investigated the cell-specific distribution of AGE-albumin in human microglial cells. The microglial marker Iba-1 was generally co-expressed with AGE and albumin in human microglial cells (HMO6, Fig. 4A). Based on these results, we concluded that AGE-albumin, the most abundant protein modified by AGE, was produced largely by microglial cells in the human brain from alcoholic individuals. Co-immunoprecipitation analyses showed dose-dependent increments in the amounts of AGE-albumin in total lysates of HMO6 cells following exposure to 0, 25, 50, or 100 mM ethanol for 24 h (Fig. 4B). Next, we evaluated whether AGE-albumin secretion was increased when human HMO6 microglial cells were treated with ethanol. Immunoblot analyses of whole cell lysates and an ELISA of culture media showed that AGE-albumin synthesis in human microglial cells and its extracellular secretion were significantly elevated up to two-fold in a dose-dependent manner after ethanol treatment (Fig. 4C). To verify whether neuronal cells synthesize MCP-1 following ethanol treatment, we measured the MCP-1 concentration after exposure to 0, 25, 50, or 100 mM ethanol treatment. MCP-1 concentration was increased dose-dependently in neuronal cell lysates (Fig. S3).

### 3. Induction of RAGE and promotion of neuronal death by AGE-albumin in the hippocampus of the binge alcohol-exposed rats

**Figure 5:**
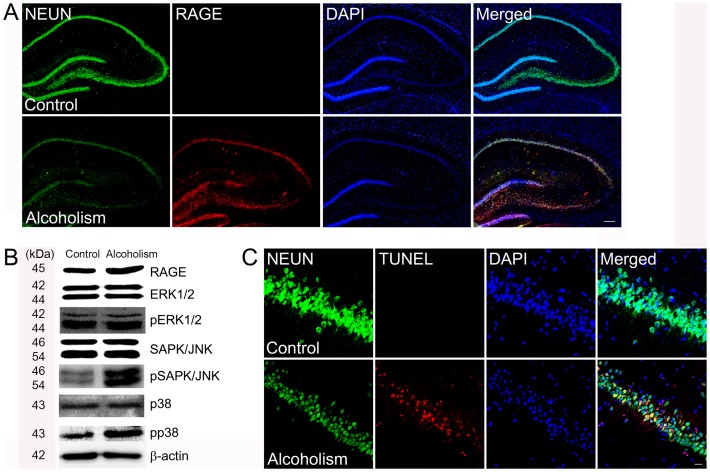
Induction of neuronal cell death by AGE-albumin through up-regulation of RAGE in the hippocampus of rats exposed to binge alcohol. (A) Relative levels of Neun (green), RAGE (red), and DAPI (blue) in the hippocampus of the control or rats exposed to binge-alcohol were evaluated by triple confocal microscopic image analyses. Scale bar  = 200 µm. (B) Immunoblot analysis was performed to determine the expressed levels of RAGE, ERK1/2, pERK1/2, SAPK/JNK, pSAPK/JNK, p38, pp38, and β-actin, used as an internal control for equal protein loading of each lane. (C) Neuronal cell apoptosis was evaluated by triple labeling with NEUN (neuron marker, green), TUNEL (apoptotic cell marker, red) and DAPI (blue). Scale bar  = 50 µm.

RAGE is expressed in neurons, and its increased level is highly correlated with neuronal death [29], [30]. Therefore, we also assessed whether AGE-albumin increased production of RAGE, a strong indicator of neuronal apoptosis in the hippocampus of the binge alcohol-exposed rats. Immunohistochemical analysis showed that RAGE level increased significantly in the hippocampus of the rats exposed to binge-alcohol compared to that in the untreated rats (Fig. 5A, B). The immunohistochemical data also showed that the number of terminal deoxynucleotidyl transferase dUTP nick end labeling (TUNEL)-positive cells, an apoptotic marker, increased significantly in the hippocampus of the binge alcohol-exposed rats compared to that in untreated animals (Fig. 5C).

Since stress-activated mitogen activated protein kinases (MAPKs) and increased mitochondrial calcium influx are critically important for initiating apoptosis [31], we monitored changes in the relative levels of RAGE and MAPKs in the hippocampus of the control and the binge-alcohol-exposed rats. Immunoblot analyses showed that the levels of RAGE, phosphorylated extracellular regulated kinase pSAPK/JNK and pp38 increased significantly in the hippocampus but not in the cerebellum of the binge-alcohol rats compared to those of controls (Figs. 5B, S4).

### 4. Protection of alcohol-mediated neuronal death by an AGE inhibitor

**Figure 6:**
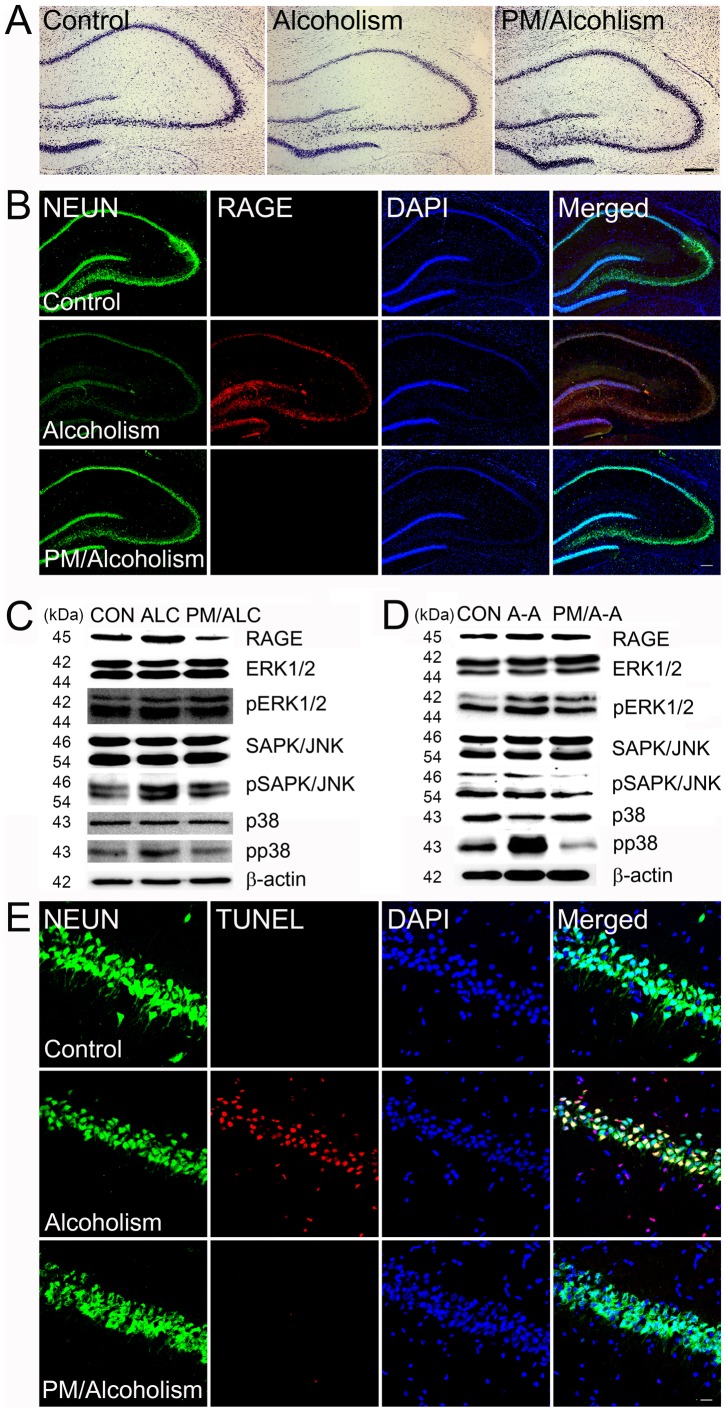
Protection of ethanol-mediated neuronal cell death by the AGE inhibitor pyridoxamine by decreasing RAGE levels. (A) Relative levels of neurons in the hippocampus of rat brains were evaluated by cresyl violet staining after alcohol and pyridoxamine (PM) co-treatment. Scale bar  = 200 µm (B) The relative levels of RAGE positive neurons with Neun (green), RAGE (red), and DAPI (blue) in the hippocampus of the control or rats exposed to bingealcohol with or without pyridoxamine co-treatment were evaluated by triple confocal microscopic image analyses. Scale bar  = 200 µm (C–D) Immunoblot analysis was conducted to evaluate the expressed levels of RAGE, ERK1/2, pERK1/2, SAPK/JNK, pSAPK/JNK, p38, pp38, and β-actin in the hippocampus of the control or binge alcohol-exposed rats with or without pyridoxamine co-treatment (C) and neuron cell lysate (D). β-actin was used as an internal control for equal protein loading of each lane (CON; Control, ALC; Alcoholism, PM/ALC; Pyridoxamine/alcoholism, A-A; AGE-Albumin, PM/A-A; Pyridoxamine/AGE-Albumin). (E) Neuronal cell apoptosis was evaluated by triple labeling with NEUN (neuron marker, green), TUNEL (apoptotic cell marker, red) and DAPI (blue) in the hippocampus of the control or rats exposed to binge alcohol with or without pyridoxamine co-treatment. Scale bar  = 50 µm.

To investigate the protective effect of an AGE inhibitor on alcohol-mediated neuronal death, we compared the number of neurons in rat brains treated with alcoholalone versus co-treated with pyridoxamine. The relative number of neurons in the hippocampus in alcohol-exposed rat brains were increased dramatically 10 days after pyridoxamine co-treatment compared to those exposed to ethanol alone (Fig. 6A). Triple labeling confocal microscopic analysis was also performed to investigate whether ethanol treatment induced neuronal death through elevated RAGE levels while pyridoxamine would protect from RAGE-mediated neuronal death. The relative number of RAGE-positive neurons increased dramatically after exposure to ethanol alone but decreased markedly after rats were co-treated with ethanol/pyridoxamine (Fig. 6B). The triple immunohistochemical results also revealed that the number of TUNEL-positive neuronal cells decreased dramatically in the hippocampus after pyridoxamine treatment compared to that in the rats treated with binge-alcohol alone (Fig. 6C).

Immunoblot analyses showed that the levels of RAGE, pSAPK/JNK, and pp38 increased significantly in the hippocampus of the rats exposed to binge-alcohol alone. However, the number of neuronal cells was decreased by treatment with ethanol alone, as compared to those in the control. However, pyridoxamine co-treatment increased the number of neuronal cells, as similar to the control rats (Fig. 6D, E). These results demonstrate that AGE-albumin produced and secreted from activated microglial cells directly promotes apoptosis of neuronal cells following binge alcohol exposure by activating the MAPK-dependent cell death pathway.

## Discussion

Many investigators have reported the direct involvement of activated microglial cells in various neurodegenerative diseases including alcohol-induced brain damage [14], [15], [20], [32], [33]. However, the mechanism by which activated microglia cells promote neuronal cell damage is poorly understood. By using immunoblot and mass-spectromeal analyses, we have reported that albumin is synthesized in microglial cells of the brain [34]. We have recently demonstrated that albumin, synthesized mainly from activated microglial cells, is conjugated with AGE to produce a potently toxic AGE-albumin, which promotes neuronal cell death in animal models of AD [28]. This conclusion is further supported by the results obtained from animal experiments as well as human brain specimens from AD individuals compared to the corresponding control people.

To study the mechanisms by which AGE-albumin synthesis increases and how it promotes neuronal cell death during alcohol over-exposure, we first investigated the respective distribution of AGE and albumin in the brain of human alcoholics. Immunohistochemical analysis revealed that most AGEs were co-localized with albumin, suggesting that AGE-albumin could be a major AGE product in microglial cells of the brains of human alcoholics. This result strongly indicates that AGE-albumin may be directly involved in cell death in the brains of alcoholics.

Subsequent immunohistochemical analysis of rat brain before and after alcohol treatment for 10 consecutive days revealed that the number of activated microglial cells increased significantly in three areas of the hippocampus, the entorhinal cortex, and the ventral tegmental area in the binge ethanol-exposed rats. Cresyl violet staining also showed that the number of neuronal cells was decreased dramatically in the three areas of the alcohol-exposed rat brains compared to those of the control. In addition, AGE and albumin tissue levels were strikingly elevated in the rats exposed to binge ethanol compared with those in control rats. These microglial aggregation and neuronal death results are exactly same as those in the previous reports [8], [16], although the role and distribution of AGE-albumin were not studied in the previous reports. Consequently, we further investigated the cell-specific distribution of AGE-albumin in human microglial cells. The microglial marker Iba-1 was generally co-expressed with AGE and albumin. Based on these results, we concluded that AGE-albumin, the most abundant protein modified by AGE, is produced largely by activated microglial cells in the rat brain after ethanol treatment.

Next, we evaluated whether AGE-albumin secretion was increased when human HMO6 microglial cells were activated by MCP-1, which is a strong microglial cell chemokine and increased after alcohol exposure. Immunoblot analysis of whole cell lysates and the ELISA data of culture media showed that AGE-albumin synthesis in human microglial cells and its extracellular secretion were significantly elevated in a dose-dependent manner after MCP-1 treatment. These results suggest that the neurons in the three specific areas are more sensitive to alcohol-induced damage than those in other brain areas, possibly through secreted cytokines such as MCP-1 to activate microglial cells. This may have contributed to the localized lesions in the three specific areas, as reported previously [6]–[8], [21]. However, we observed neuronal degeneration in other areas of the brain in the binge long-term ethanol model. This observation could also explain that long-term heavy alcohol ingestion can damage many neuronal cells in the brain, but that the three areas are more sensitive to damage through elevated levels of secreted AGE-albumin.

RAGE is expressed in neurons and its increased level is highly correlated with neuronal death [35]–[37]. Therefore, we also evaluated whether AGE-albumin increases the production of RAGE, a strong indicator of neuronal apoptosis in the hippocampus, entorhinal cortex, and ventral tegmental areas in rats exposed to a binge ethanol exposure model. The immunohistochemical data showed that the amount of RAGE increased significantly in the three areas of the binge-alcohol rats compared to untreated control animals. Thus, severe loss of neurons in the three areas also seemed to be triggered by the AGE-RAGE interaction.

The intracellular mechanism of neuronal death after activating RAGE is important to understand the pathology and for identifying potential therapy targets for alcohol-mediated neuronal death and neurodegeneration. Since the stress-activated MAPKs are critically important for initiating apoptosis [31], [38]–[40], we also monitored the changes in the respective levels of MAPKs and cell death rate in human primary neurons (SHSY5Y). Immunoblot analyses showed that the levels of pSAPK/JNK and pp38 were increased significantly in the binge alcohol-exposed rats compared to those in the control. These results are consistent with death of neuronal cells in the hippocampus, entorhinal cortex, and ventral tegmental areas in rats exposed to the binge-ethanol. Similar to the case of AD [28], the MAPK-dependent apoptosis pathway appears to play a key role in promoting ethanol-induced neurotoxicity.

We also studied the beneficial effect of an AGE-albumin inhibitor pyridoxamine on ethanol-mediated neuronal death in rats. The relative number of neurons in the rat brain was dramatically increased 10 days after pyridoxamine co-treatment compared to that of rats exposed to ethanol alone. Triple labeling confocal microscopy revealed that the relative levels of brain AGE, albumin- and Iba1-positive cells were significantly increased in the ethanol-treated rats, but decreased in the ethanol- and pyridoxamine-co-treated rats. A triple labeling confocal microscopic analysis was also performed to investigate whether ethanol treatment induces neuronal death and pyridoxamine protects against RAGE-mediated neuronal death. The relative numbers of RAGE-positive neurons dramatically increased by ethanol alone but decreased markedly after ethanol/pyridoxamine co-treatment. The numbers of pp38 and pSAPK/JNK-positive neurons were increased markedly after treatment with ethanol alone but decreased in ethanol/pyridoxamine co-treated rats.

These data demonstrate that AGE-albumin produced from activated microglial cells directly promotes apoptosis of neuronal cells following binge alcohol treatment by activating the pSAPK/JNK, pp38-dependent apoptosis pathway, as demonstrated previously in different cell types [28].

In summary, our current data show that AGE-albumin, the most abundant form of brain AGE, is synthesized in activated microglial cells and secreted into the extracellular space following binge alcohol exposure. Elevated levels of AGE-albumin are frequently observed in human brains of alcoholic individuals compared with those in control people. Furthermore, AGE-albumin promotes the pSAPK/JNK-pp38 dependent apoptosis in human neurons. Treatment with an AGE inhibitor pyridoxamine significantly protected neurons from apoptotic death in an animal model of binge alcohol exposure. Our results provide novel mechanistic insights by which microglial cells play an important role in promoting neuronal death in alcohol-exposed rats by synthesizing and secreting potentially toxic AGE-albumin. Finally, AGE-albumin could be an excellent biomarker and a therapeutic target for alcohol-induced neuronal death and possibly dementia.

## Supporting Information

Figure S1 **Distribution of activated microglial cells and neuron in the entorhinal cortex (ENT), ventral tegmental area (VTA), and periaqueductal gray matter (PAG) of control and the binge-alcohol animal model.**
(DOCX)

Figure S2 **AGE-albumin level in hippocampus of rat brains showed by single channel.**
(DOCX)

Figure S3 **Increased synthesis of MCP-1 in human neuronal cells.**
(DOCX)

Figure S4 **Relative levels of RAGE and MAPK in cerebellum.**
(DOCX)

Table S1 **List of experiment number in vitro study.**
(DOCX)

Table S2 **List of experiment number in vivo study.**
(DOCX)
