# Supplementary material for: Microglial AGE-Albumin Is Critical in Promoting Alcohol-Induced Neurodegeneration in Rats and Humans
Source: PLoS One. 2014 Aug 20;9(8):e104699. doi: 10.1371/journal.pone.0104699 (PMC4139297; doi:10.1371/journal.pone.0104699)
Supplement: Figure S1 — Distribution of activated microglial cells and neuron in the entorhinal cortex (ENT), ventral tegmental area (VTA), and periaqueductal gray matter (PAG) of control and the binge-alcohol animal model. (DOCX) [file pone.0104699.s001.docx]

**Figure S1.** Distribution of activated microglial cells and neuron in the entorhinal cortex (ENT), ventral tegmental area (VTA), and periaqueductal gray matter (PAG) of control and the binge-alcohol animal model.

(A) OX-42-positive microglial cells were measured by immunohistochemical staining in control and time-dependent brain tissues on days 9 and 11 after alcohol treatment. (B) Cresyl violet staining was used to detect cell number in the ENT, VTA and PAG of alcoholic rat brain on days 9 and 11. Scale bar = 200 μm.
