# Supplementary material for: Microglial AGE-Albumin Is Critical in Promoting Alcohol-Induced Neurodegeneration in Rats and Humans
Source: PLoS One. 2014 Aug 20;9(8):e104699. doi: 10.1371/journal.pone.0104699 (PMC4139297; doi:10.1371/journal.pone.0104699)
Supplement: Figure S2 — AGE-albumin level in hippocampus of rat brains showed by single channel. (DOCX) [file pone.0104699.s002.docx]

**Figure S2.** AGE-albumin level in hippocampus of rat brains showed by single channel.

Triple-labeled confocal microscopic image analyses were used to study the distribution and relative levels of albumin (green), AGE (red), and DAPI (blue) in the hippocampus Cornu Ammonis Area1 (CA1), Cornu Ammonis Area2 (CA2), Cornu Ammonis Area3 (CA3), Dendate Gyrus (DG) of the control rats (A) and binge-alcohol exposed rats (B). Scale bar = 50 μm.
