# Supplementary material for: Microglial AGE-Albumin Is Critical in Promoting Alcohol-Induced Neurodegeneration in Rats and Humans
Source: PLoS One. 2014 Aug 20;9(8):e104699. doi: 10.1371/journal.pone.0104699 (PMC4139297; doi:10.1371/journal.pone.0104699)
Supplement: Figure S3 — Increased synthesis of MCP-1 in human neuronal cells. (DOCX) [file pone.0104699.s003.docx]

**Figure S3.** Increased synthesis of MCP-1 in human neuronal cells.

The dose-dependent changes of MCP-1 were determined in cell lysates of SH-SY5Y cells control and treated with 25, 50 or 100 mM ethanol for 24 h by RT-PCR. GAPDH in the cell lysate internal control for equal mRNA loading of each lane.
