# Supplementary material for: Microglial AGE-Albumin Is Critical in Promoting Alcohol-Induced Neurodegeneration in Rats and Humans
Source: PLoS One. 2014 Aug 20;9(8):e104699. doi: 10.1371/journal.pone.0104699 (PMC4139297; doi:10.1371/journal.pone.0104699)

**Figure S4**. Relative levels of RAGE and MAPK in cerebellum.

Immunoblot analysis was used for protein expression levels of RAGE, ERK1/2, p38, SAPK/JNK, pERK1/2, pp38, pSAPK/JNK, and β-actin in the cerebellum internal control for equal protein loading of each lane.


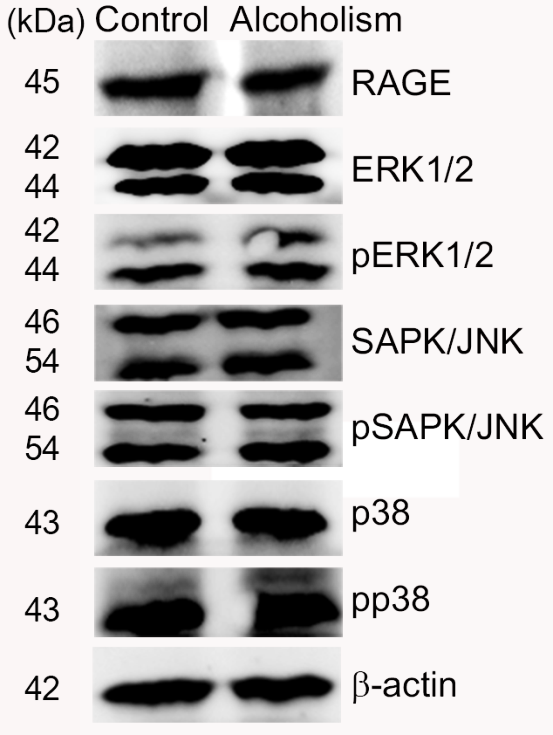

Supplement: Figure S4 — Relative levels of RAGE and MAPK in cerebellum. (DOCX) [file pone.0104699.s004.docx]
