# Supplementary material for: Microglial AGE-Albumin Is Critical in Promoting Alcohol-Induced Neurodegeneration in Rats and Humans
Source: PLoS One. 2014 Aug 20;9(8):e104699. doi: 10.1371/journal.pone.0104699 (PMC4139297; doi:10.1371/journal.pone.0104699)
Supplement: Table S2 — List of experiment number in vivo study. (DOCX) [file pone.0104699.s006.docx]

**Table S2.** List of experiment number in vivo study

|  | Experiment list | Animal/section | Trail number |
| --- | --- | --- | --- |
| 1 | Cresyl violet staining | 3/3 | 5 |
| 2 | Immunostaining | 3/3 | 5 |
| 3 | Co-Immunoprecipitation | 3/1 | 5 |
| 4 | Immunoblot | 3/1 | 5 |
| 5 | TUNEL staining | 3/3 | 5 |
